# Analysis of the Reversible Impact of the Chemodrug Busulfan on Mouse Testes

**DOI:** 10.3390/cells10092403

**Published:** 2021-09-13

**Authors:** Laura Thirouard, Hélène Holota, Mélusine Monrose, Manon Garcia, Angélique De Haze, Jean-Paul Saru, Françoise Caira, Claude Beaudoin, David H. Volle

**Affiliations:** INSERM U1103, Université Clermont Auvergne, CNRS UMR-6293, GReD Institute, Team-Volle, F-63001 Clermont-Ferrand, France; laura.thirouard@uca.fr (L.T.); Helene.HOLOTA@uca.fr (H.H.); Melusine.MONROSE@uca.fr (M.M.); Manon.GARCIA@uca.fr (M.G.); Angelique.DE_HAZE@uca.fr (A.D.H.); J-Paul.SARU@uca.fr (J.-P.S.); Francoise.CAIRA@uca.fr (F.C.)

**Keywords:** spermatogonial stem cell, chemotherapy, depletion, regeneration and fertility

## Abstract

Spermatogenesis is a process within the testis that leads to the production of spermatozoa. It is based on a population of spermatogonial stem cells, which have the capacity to self-renew and to differentiate throughout life to ensure the functions of reproduction are maintained. Male fertility disorders are responsible for half of the cases of infertility in couples worldwide. It is well known that cancer treatments are associated with reversible or irreversible fertility disorders. Busulfan (Bu) is an alkylating agent that significantly inhibits spermatogenesis. The present study relied on a combination of in vivo and in vitro approaches as well as RNAseq analysis to characterize the effects of Bu, in which mouse testes were used as a model. An in silico analysis revealed that many of the Bu-modulated genes are potentially regulated by the SIN3 Transcription Regulator Family Member A (SIN3A) and E2F Transcription Factor (E2F) families of transcription factors. The results demonstrate that the deregulated genes function in processes related to the cell cycle, DNA repair, and cell death mechanisms, including the Tumor Protein 53 (TP53) pathway. This reinforces the role of the TP53 signaling pathway as a major player in Bu effects. In addition, Bu altered the patterns of mRNA accumulation for various genes in undifferentiated spermatogonia. This work provides significant insight into the kinetics and impacts of busulfan, which could pave the way for developing strategies to minimize the impact of chemodrugs and, thus, could lead to germ cell lineage regeneration following anticancer treatments.

## 1. Introduction

Spermatogenesis is the process of spermatozoa production. In mammals, spermatogenesis takes place in the testicles beginning at puberty and is constantly maintained throughout life. The duration of spermatogenesis is 35 days in mice and 74 days in humans, and the process is subdivided into several stages, each with a defined duration. The maintenance of spermatogenesis requires spermatogonial stem cells, which self-renew throughout life. These spermatogonial stem cells (*SSC*s) then enter a process of proliferation and differentiation before meiosis is initiated and they become spermatozoa, which are released into the lumen of seminiferous tubules.

For several decades, there has been a steady increase in fertility disorders, which currently affect 10–15% of couples worldwide [1,2]. For 60% of men with fertility problems, the defects correspond to an alteration in the production of spermatozoa by the testicle [3]. Cancer therapies, such as chemotherapy, are known to have significant negative effects on male reproductive function, leaving many men permanently infertile due to changes in spermatogenesis [4,5]. There are many different chemotherapy drugs, administered intravenously or orally, that can be used alone or in combination to treat a wide variety of cancers.

Predicting long-term reproductive outcomes of cancer treatments in cancer survivors based on the specific chemotherapy regimen may improve pretreatment fertility preservation counseling and future reproductive outcomes. Alkylating agents are the chemotherapy molecules with the greatest impact on germ cell lineage. Indeed, alkylating agents have been shown to cause long-term depletion of the spermatogonial pool [6].

Mouse rodent models have been effectively used to better decipher the impact of chemodrugs on male fertility by evaluating their effects on male gonads and, more specifically, on germ cell lineage. Although a combination of chemotherapy drugs is widely exploited to improve cancer treatment in clinical settings, here, we analyzed the impact of a single drug, namely, busulfan (Bu). Busulfan is an alkylating anticancer agent that preconditions for hematopoietic stem cell transplantation for the treatment of chronic myeloid leukemia. It acts preferentially by adding an alkyl group between two guanines of the DNA or between a guanine and an adenine. The addition of this group results in intra-strand bridge formation in the DNA leading to single-strand breaks that block DNA replication and transcription. In the long term, the inhibition of cell proliferation and differentiation was observed [7]. It has been demonstrated that exposure to chemotherapy leads to testicular damage with germ cell loss. High doses of chemotherapy may lead to complete depletion of the seminiferous tubules, resulting in permanent sterility. At lower doses, germ cell recovery has been observed, as has been documented for busulfan exposure. This work provides insight into the kinetics and impacts of busulfan that could pave the way for developing strategies to minimize the impact of chemodrugs and thus to allow for progression of the germ cell lineage, resulting in regeneration and recolonization of the seminiferous epithelium.

## 2. Materials and Methods

### 2.1. Animals

The mice used in this study were based on the C57BL/6J background (Charles River, abresle, France).

Mice were housed in temperature-controlled rooms with 12 h light/dark cycles. Mice had ad libitum access to food and water. The refinement was based on the housing and monitoring of the animals as well as the development of protocols that take into account animal welfare. This was achieved by enriching the cages with cardboard tunnels and mouse houses. The mice were housed in social groups, with cage sizes that complied with the number of mice according to the legislation.

In the experimental design, two groups of 12-week-old mice were used—C57BL/6J males treated with vehicle (DMSO, Sigma-Aldrich, L’Isle d’Abeau, France) or busulfan (Bu; 15 mg/kg) (Sigma-Aldrich, L’Isle d’Abeau, France). The animals were treated only once, at the beginning of the experiment, with a single IP injection of 100 µL. The mice were subsequently sacrificed at different time points. The organs were harvested at determined times after busulfan exposure.

To determine the number of animals to use, we previously conducted experiments to investigate Bu-induced testicular damage [8,9]. Busulfan treatment was expected to be effective if it changed the value of analyzed parameters by 20%, and the results were analyzed with *p* < 0.05 considered to indicate a statistically significant difference. The calculation for the number of animals required to establish statistical significance of the results found was a minimum of *n* = 16 animals per group.

This study was conducted in accordance with the current regulations and standards approved by Institut National de la Santé et de la Recherche Médicale Animal Care Committee and by the animal care committee (CEMEA Auvergne; protocol CE 07–12 (19 December 2012) and then renewed as APAFIS #: 19626-2020072312102562v3 (4 January2021)).

### 2.2. Fertility Test

The fertility tests started 6 weeks after treatment and continued for the following 15 days. Each male (*n* = 16 per group) was mated with a female C57BL/6J (Charles River, abresle, France) at night, and the males were removed from the female’s cage each day. Vaginal plugs were monitored every morning at 8:00 a.m. After 14 days, the mating efficiency was inspected and the numbers of pups per litter were counted.

### 2.3. Histology

The testes were collected, fixed in 4% PFA, and embedded in paraffin. Then, 5 μm thick sections were prepared and stained with H&E (*n* = 6–10 animals per group).

### 2.4. In Vivo TUNEL Analysis

Terminal deoxynucleotidyl transferase dUTP nick end labeling (TUNEL) experiments were performed as previously described [8] on 5 μm sections of a testis fixed in 4% paraformaldehyde. In each testis, at least 100 random seminiferous tubules were counted from at least 2 independent sections, and all the seminiferous tubules were analyzed for the counting. The results are expressed as the number of TUNEL-positive cells per seminiferous tubule.

### 2.5. Immunohistochemistry

In the next step, 5 μm sections were mounted on positively charged glass slides (Superfrost plus), and then deparaffinized, rehydrated, treated for 20 min at 93–98 °C in 0.01 M citric buffer–Tween 0.1% (pH 6), rinsed in osmosed water (2 × 5 min), and washed (2 × 5 min) in phosphate-buffered saline (PBS). Immunohistochemical studies were conducted according to the manufacturer’s recommendations. The slides were then counterstained with Hoechst medium (1 mg/mL) (Invitrogen, Cergy Pontoise, France) and mounted on PBS/glycerol (50/50). For the histological and immunohistological analysis of each section, all of the seminiferous tubules were counted. In each testis, at least 100 random seminiferous tubules were counted from at least 2 independent sections.

### 2.6. Cell Line Approaches

GC2spd (ts) cells were obtained from ATCC (CRL-2196). GC1spg cells were used as previously described [8]. Then, 24 h after plating, the cells were starved for 12 h and treated with either DMSO or busulfan (200 µM).

### 2.7. Real-Time RT-PCR (qPCR)

The RNA from the GC1spg samples were isolated using RNAzol-RT (Sigma-Aldrich, L’Isle d’Abeau, France). cDNA were synthesized from the total RNA with MMLV and random hexamer primers (Promega, Charbonnières-les-Bains, France). The real-time PCR measurement of individual cDNA was performed using SYBR green dye (Master mix Plus for SYBR Assay, Takara Bio Inc, Shiga, Japan) to measure the duplex DNA formation with the Eppendorf Realplex system. For each experiment, standard curves were generated with pools of cDNA from cells with different treatments. The results were analyzed using the ΔΔct method, and the primer sequences were used previously [8,10,11,12].

### 2.8. RNASeq

The experiment was performed on GC1spg cells treated for 24 h with DMSO or busulfan (200 µM). Starting with the RNA, all preparations were made using the genomeast IGBMC platform (Illkirch). The mRNAseq libraries were sequenced (1 × 50 b).

Reads were mapped onto the mm10 assembly of the mouse genome using Hisat2 v2.1.0 [13] and the BoWtie2 v2.1.0 aligner [14]. Only uniquely aligned reads were retained for further analysis.

The gene expressions were quantified using HTSeq v0.5.4p3 [15] with gene annotations from Ensembl release 77.

Read counts were normalized across libraries with the method proposed by Anders and Huber [16]. The groups were compared using the method previously described [17] and implemented in the DESeq2 Bioconductor library (DESeq2 v1.0.19). The resulting *p*-values were adjusted for multiple tests using the method of Benjamini and Hochberg [18].

We generated lists of genes (FC > 1.25 and *p* < 0.01) that were differentially expressed after Bu exposure.

The accession number for the RNAseq data reported in this paper is GSE164734.

### 2.9. Statistical Analyses

The number and type of replicates (e.g., technical replicates, independent experiments, number of mice, and number of independent litters) are reported in the figure legends. Error bars represent the SEM. Differences between the groups were determined by ANOVA. All numerical data are represented as the mean ± SEM. The level of significant difference was set at *p* < 0.05.

## 3. Results and Discussion

**Figure 1:**
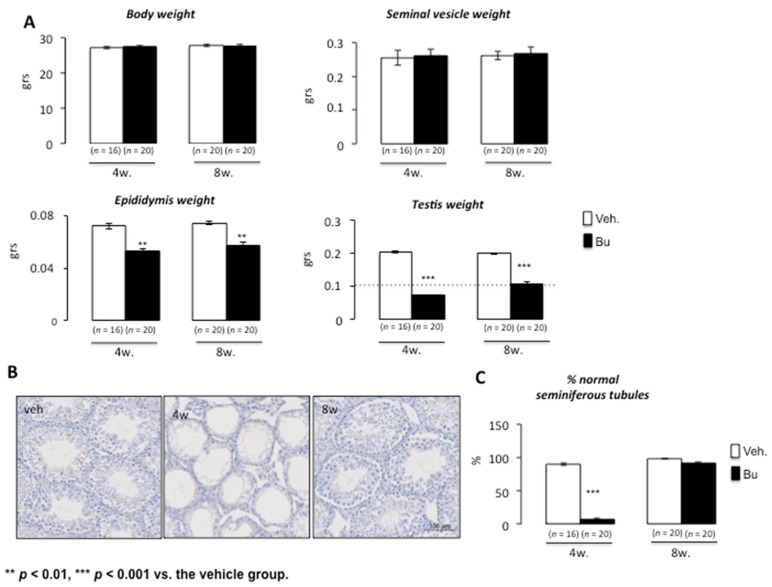
Acute busulfan exposure alters testes in male mice. (**A**) Body, seminal vesicle, epididymis, and testis weights of C57BL/6J males 4 or 8 weeks after busulfan or vehicle treatments. (**B**) Representative micrographs of hematoxylin/eosin-stained testes of C57BL/6J males 4 or 8 weeks after busulfan or vehicle treatments. (**C**) The number of normal seminiferous tubules of C57BL/6J males treated with the vehicle or busulfan (4 or 8 weeks after treatment). For all panels, the data were obtained from at least three independent experiments; the number of animals per group is indicated in brackets below the bars of the graphs. The data are expressed as the means ± SEM. Statistical analysis: ** *p* < 0.01, and *** *p* < 0.001 vs. the vehicle group. Veh.: vehicle and Bu: busulfan.

**Figure 2:**
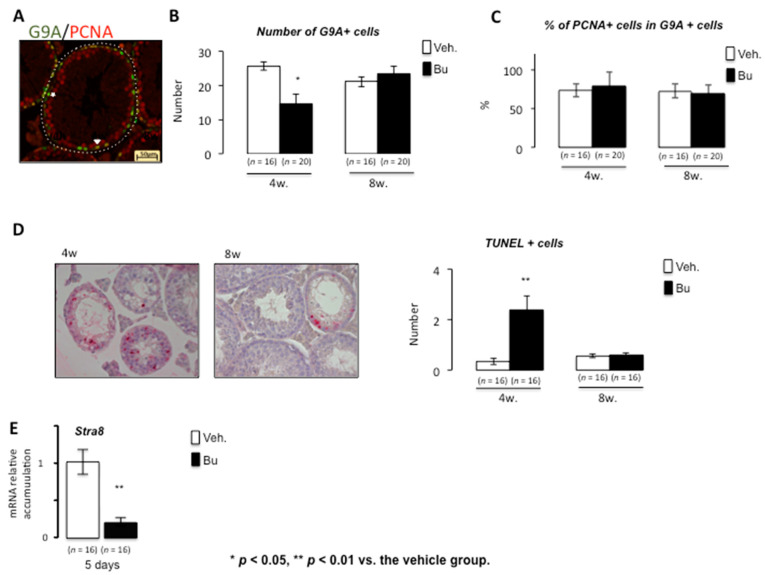
Acute busulfan exposure alters spermatogenesis and germ cell survival in male mice. (**A**) Representative micrograph of vehicle- or Bu-treated testis stained for G9A (green) and PCNA (red). The arrowhead indicates G9A/PCNA-positive cells, and the star indicates PCNA-negative and G9A-positive cells. (**B**) The number of G9A-positive cells per seminiferous tubules in testes treated with vehicle or busulfan (4 and 8 weeks after treatment). (**C**) The percentage of G9A-positive cells co-stained for PCNA in testes treated with vehicle or busulfan (4 and 8 weeks after treatment). (**D**) (Left) Representative micrograph of testes of vehicle- or Bu-treated males stained for TUNEL. (Right) The number of TUNEL-positive cells per seminiferous tubule of males treated with vehicle or busulfan (4 or 8 weeks after treatment). (**E**) Relative *Stra8* mRNA accumulation normalized to β-actin on testis after exposure to vehicle or Bu after 5 days. For all panels, the data were obtained from at least three independent experiments; the number of animals per group is indicated in parentheses below the bars of the graphs. The data are expressed as the means ± SEM. Statistical analysis: * *p* < 0.05, ** *p* < 0.01 vs. the vehicle group. Veh.: vehicle and Bu: busulfan.

Busulfan has transitory effects on germ cell lineage at low doses. It has been demonstrated that exposure to chemotherapy drugs leads to testicular damage with germ cell loss. High doses of chemotherapy may lead to complete depletion of the seminiferous tubules, resulting in permanent sterility. At lower doses, germ cell recovery was observed; this has been documented in relation to busulfan exposure. Rodent experimental models were used, and the exposure to busulfan was well controlled, allowing for germ cell reemergence, which led to fertility recovery [19]. Using a dose compatible with germ cell recovery (15 mg/kg), our results show that, after busulfan exposure, there was no impact of busulfan on body weight at 4 and 8 weeks compared with vehicle-treated animals (Figure 1A). Regarding the urogenital tract, if no effect of Bu was noticed on the weights of seminal vesicles, a decrease in the epididymis weight was observed at both 4 and 8 weeks after Bu exposure compared with the control animals (Figure 1A). Interestingly, the testicular weight was reduced at 4 weeks and then increased from this point, as observed at 8 weeks (Figure 1A), suggesting an ability of germ cells to regenerate testicular epithelium over time. This was correlated with the analysis of the percentage of seminiferous tubules with altered or almost normal epithelium at 4 and 8 weeks after exposure, as illustrated by H&E staining, which showed a huge loss in germ cells at 4 weeks and recovery by 8 weeks (Figure 1B,C). In addition, our results show that Bu exposure led to a decrease in the number of spermatogonia/spermatocytes, as revealed by G9A immunostaining at 4 weeks (Figure 2A). The number of G9A-positive cells increased back to almost normal levels at 8 weeks (Figure 2B). This impact on germ cells was not associated with altered proliferation, as highlighted by the percentage of positive PCNA on G9A-stained cells (Figure 2C). The significant germ cell loss, mainly observed 4 weeks after treatment, was correlated with an increase in apoptotic germ cells (Figure 2D). The retinoid pathway is demonstrated to alter germ cell production and can lead to meiotic default associated with apoptosis [20]. Our results show that, at 5 days following exposure, Bu led to decreased mRNA accumulation of *Stra8*, a retinoid target gene (Figure 2E). The lower expression of *Stra8* (Stimulated by retinoic acid gene 8), a gene known to be involved in meiosis entry and progression [21], suggests that Bu could be associated with impaired vitamin A homeostasis, explaining some of the observed testicular phenotypes.

**Figure 3:**
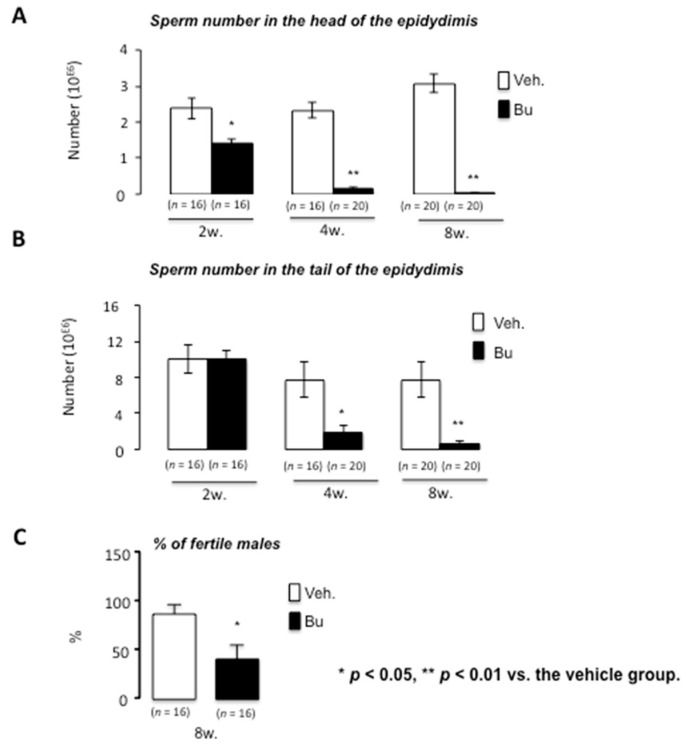
Busulfan alters germ cell production and male fertility in mice. (**A**) Sperm count in the epididymis head of C57BL/6J males at 2, 4, and 8 weeks after busulfan or vehicle treatments. (**B**) Sperm count in the epididymis tail of C57BL/6J males at 2, 4, and 8 weeks after busulfan or vehicle treatments. (**C**) Percentage of fertile males 8 weeks after vehicle or busulfan treatment following breeding with C57BL/6J females. For all panels, the data were obtained from at least the independent experiments; the number of animals per group is indicated in parentheses below the bars of the graphs. The data are expressed as the means ± SEM. Statistical analysis: * *p* < 0.05, ** *p* < 0.01 vs. the vehicle group. Veh.: vehicle and Bu: busulfan.

These results for testicular histology were supported by analysis of the number of sperm cells produced, as revealed by the count of sperm cells in the head and tail of the epididymis at both 4 and 8 weeks (Figure 3A,B). Interestingly, the impact was first noticed at the head of the epididymis 2 weeks after Bu exposure, clearly illustrating the main impact of Bu on spermatogenesis rather than on epididymis (Figure 3A,B). As expected, Bu treatment was associated with a significant and progressive decrease in sperm cell production at 4 and 8 weeks after treatment, which is consistent with the fact that the testis did not recover its complete structure by 8 weeks. This was associated with a decrease in the percentage of fertile males 8 weeks following Bu exposure (Figure 3C). Indeed, if 80% of vehicle-treated males were fertile, less than 50% were able to provide progeny in the Bu-treated group.

Analyzing the impacts of Bu on mouse testes is quite complicated because it results in a strong modulation of the representation of different testicular cell types. Thus, at 4 weeks, molecular assays were previously used to track germ cell loss and the reciprocal enrichment of somatic cells [8,9], resulting in an artificial increase in the mRNA accumulation of genes expressed in somatic cells (Leydig cells: *Lhcgr*, or Sertoli cells: *Fshr*) and a decrease in genes expressed in the germ cell lineage (Oct3/4, *G9a*, *Ccna1*, and *Smad6*). Then, a previously published analysis of gene expression kinetics at 8 weeks showed that they reflected the germ cell recovery, returning to an almost normal testicular cell composition and, thus, showing a return to normal expression patterns compared with the vehicle-treated mice (see our previously published work [8,9]).

As Bu exposure led to cellular modulation, we deciphered the effects of Bu using cell line approaches. We analyzed the impact of Bu on two cell lines, the GC1spg and the GC2spd (ts), which represent the spermatogonia/spermatocytes and spermatocytes, respectively, and which are the cell populations impacted by Bu and almost completely lost in mouse testes 4 weeks after the treatment.

**Figure 4:**
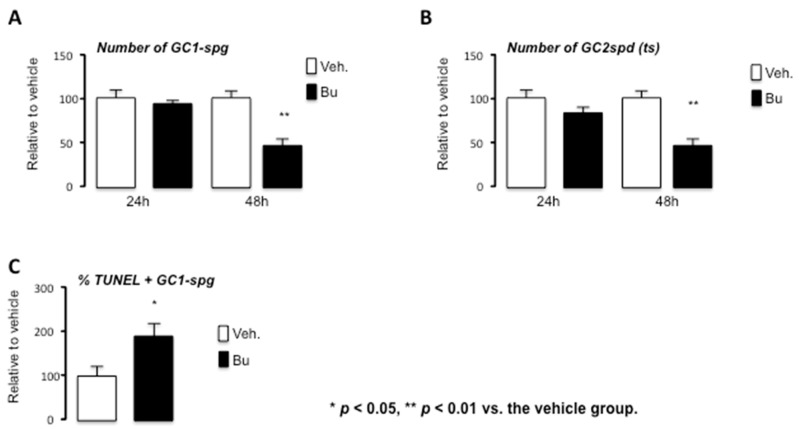
Busulfan treatment leads to the alteration of germ cell numbers through apoptosis in the GC1spg cell line. (**A**) Number of adherent cells after 24 or 48 h of treatment with vehicle or Bu in GC1spg cells. (**B**) Number of adherent cells after 24 or 48 h of treatment with vehicle or Bu in GC2spd (ts) cells. (**C**) The relative number of TUNEL-positive of vehicle- or Bu-treated GC1spg cells after 24 h. For all panels, the data were obtained from at least three independent experiments. The data are expressed as the means ± SEM. Statistical analysis: * *p* < 0.05, ** *p* < 0.01 vs. the vehicle group. Veh.: vehicle and Bu: busulfan.

A decrease in the number of GC1spg and GC2spd (ts) cells was observed 48 h after treatment with Bu (Figure 4A,B). This is consistent with the in vivo histological analysis, which highlighted that the first impact of Bu was noticed on spermatogonia, as revealed by PLZF staining starting within the first week after exposure (Supplementary Figure S1). We then decided to focus on GC1spg cells, which were more representative of PLZF + cells. A higher level of apoptotic cells was observed 24 h after treatment with Bu compared with the control cell group (Figure 4C). This could explain the decrease in the number of adherent cells 48 h after Bu exposure (Figure 4C).

**Figure 5:**
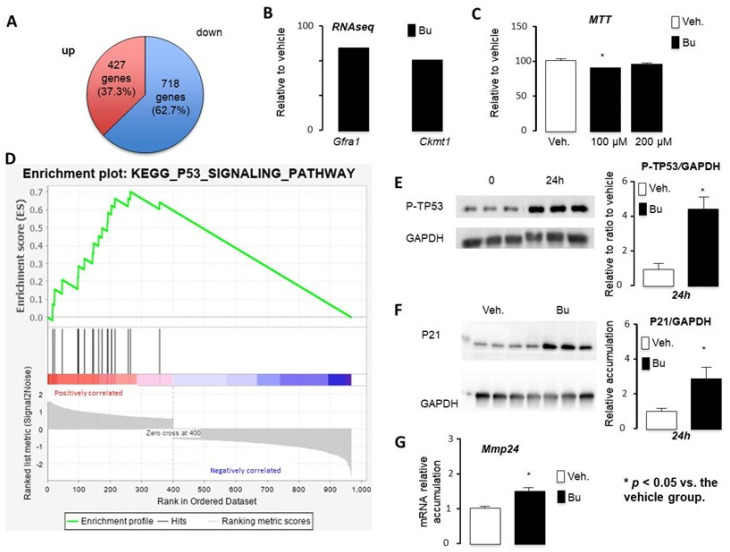
The RNAseq approach defined key targets of busulfan in the GC1spg cell line. (**A**) Venn diagram for differentially expressed genes in GC1spg cells tr eated for 24 h with vehicle or 200 μM Bu. (**B**) Fold change of *Gfra1* and *Ckmt1* obtained from the RNAseq data. (**C**) MTT data for the GC1spg cells treated for 24 h with vehicle or 100 μM or 200 μM Bu. (**D**) GSEA data obtained for genes differentially expressed in Bu- vs. vehicle-treated GC1spg cells. (**E**) Representative Western blots of GAPDH and Phospho-TP53 (P-TP53) and quantification of ratios in GC1spg cells treated with vehicle or Bu for 24 h. (**F**) Representative Western blots of GAPDH and P21 and quantification of ratios in GC1spg cells treated with vehicle or Bu for 24 h. (**G**) Relative *Mmp24* mRNA accumulation normalized to β-actin on GC1spg cells treated for 24 h with vehicle or 200 μM of Bu. Veh.: vehicle and Bu: busulfan. * *p* < 0.05, vs. the vehicle group. Veh.: vehicle and Bu: busulfan.

To further analyze the impact of Bu, GC1spg cells were exposed to the vehicle or 200 μM Bu for 24 h. Of the deregulated genes, 37.3% were upregulated (427/1145) (Table S1) and 62.7% (718/1145) were suppressed (Table S2) by Bu compared with the control group (Figure 5A). Based on the *p*-value analysis, the RNAseq results show that the most deregulated gene was *Cdk1*, the cyclin-dependent kinase, highlighting the impact on the cell cycle. Among the genes downregulated by Bu in GC1spg cells, several genes were master genes of undifferentiated germ cells, such as GDNF Family Receptor alpha 1 (*Gfra1*) (Figure 5B). The RNAseq results show that the creatine kinase-1 (*Ckmt1*) was downregulated by Bu (Figure 5B). Depletion of *Ckmt1* induces mitochondrial depolarization and apoptotic cell death. Consistently, the Bu exposure of GC1spg cells was associated with a decrease in mitochondrial activity, as reflected by MTT experiments (Figure 5C).

Moreover, even if we had not identified this pathway in our in silico analysis of the RNAseq data, it was described that an increase in lipid peroxidation was observed in busulfan-treated testes after 1–2 weeks [22], suggesting an increase in reactive oxygen species (ROS) production. Indeed, mice treated with melatonin after busulfan injection showed enhanced spermatogenesis [23], and melatonin drives the expression of MnSOD (manganese superoxide dismutase), which counteracts apoptosis caused by high levels of busulfan-induced ROS [24].

The list of genes affected by Bu was analyzed for overrepresentation analysis of gene ontology (GO) using Panther software (Table S3). GO analysis revealed enrichment in genes related to the cell cycle through alterations in microtubules and involved in DNA damage-associated processes [8].

The analysis of the Bu-altered genes, using Cistarget, revealed the SIN3A and E2F families as potential regulators of a high proportion of these genes (see Tables S4 and S5).

Of the deregulated genes, 29.5% showed potential regulation by Swi-independent chromatin modifier 3a (SIN3A). SIN3A influences gene expression during development and differentiation through various transcription factors and in a cell-specific manner. SIN3A is required for mitotic re-entry of gonocytes. Indeed, genetic inactivation of *Sin3A* in the male germline leads to infertility, resulting from the early and penetrating apoptotic death observed in germ cells lacking SIN3A and coinciding with re-entry into mitosis.

The E2F family is a potentially important regulator, as illustrated by E2F4, which targets 250 out of the 1145 deregulated genes (around 32%). The E2F transcription factors are primarily implicated in the regulation of entry and exit from the cell cycle. It has been proposed that E2F families might interact to control the maintenance of testicular tissue organization and the entry of undifferentiated quiescent spermatogonial cells into the mitotic proliferation, leading to meiosis and differentiation in the spermatozoa. Interestingly, E2F6 has been shown to form a complex with the Polycomb (PcG) group protein, which has a well-established role in gene silencing. RB/E2F and TP53 are intimately connected, and crosstalk between these pathways is critical for the induction of cell cycle arrest or cell death in response to cellular stresses. Along that line, GSEA analysis revealed the enrichment of genes associated with the TP53 pathway (Figure 5D). The analysis of KEGG pathways from our RNAseq data, consistent with previously published results, highlighted the main role of TP53 in the impact of Bu. The toxic effect of Bu is preferentially exerted on G1 phase cells [25]. Busulfan-induced DNA damage results in an increased expression of Tp53, leading to cell apoptosis [26]. The TP53 pathway activates *BAX* (Bcl2-associated X) and the expression of target genes, such as *Bax*, *Bid* (BH3-interacting domain death agonist), or *Puma* [27]. The induction of these genes allows for an increase in the permeability of the mitochondrial membrane, leading to the release of cytochrome c and the induction of apoptosis. Consistently, the level of phosphorylated TP53 was increased after Bu exposure (Figure 5E). Moreover, a 2.5-fold increase of P21 protein accumulation, a known target of TP53, was observed following Bu exposure compared with the control group (Figure 5F). In addition, the role of TP53 in the impact of Bu was validated by qPCR through the analysis of a known TP53 target, namely *Mmp24*, which was affected, according to the RNAseq results, and validated using qPCR (Figure 5G). These results highlight the main role TP53 plays in busulfan-induced effects.

**Figure 6:**
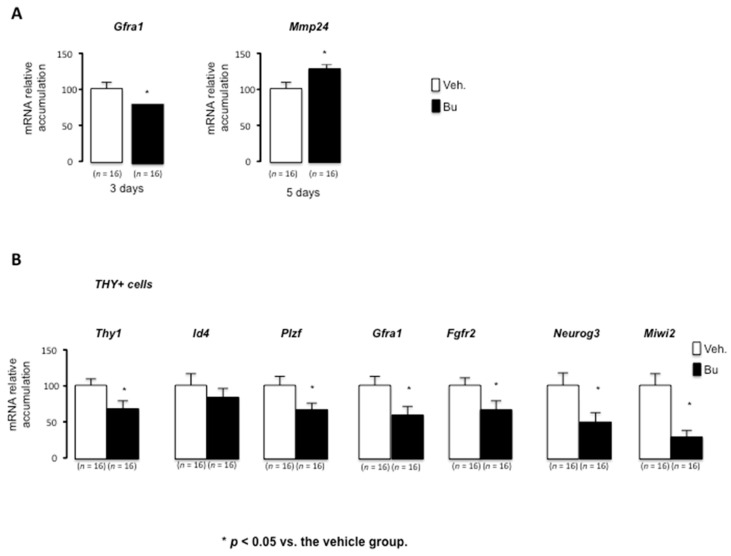
Busulfan alters key genes of undifferentiated spermatogonia in male mice. (**A**) Relative *Gfra1* and *Mmp24* mRNA accumulation normalized to *β-actin* on testes after exposure to vehicle or Bu after 3 or 5 days following exposure. (**B**) Relative *Thy1*, *Id4*, *Plzf*, *Gfra1*, *Fgfr2*, *Neurog3*, and *Miwi2* mRNA accumulation normalized to *β-actin* on FACS cell-sorted Thy1+ cells 1 week after exposure to vehicle or Bu. The data are expressed as the means ± SEM. Statistical analysis: * *p* < 0.05 vs. the vehicle group. Veh.: vehicle and Bu: busulfan.

To validate some of the results from the in vitro studies on GC1spg cells, we analyzed the expression of some genes by qPCR on the whole testis after a short period when the impact on cellularity was not of importance. The results show that Bu led to a decrease in *Gfra1* at 3 days after the treatment (Figure 6A). In addition, 5 days after the exposure to Bu, there was an increase in the TP53 target gene *Mmp24* (Figure 6A).

Treatment with busulfan induces prolonged azoospermia, which is associated with the major impacts of the treatment on spermatogonia. Indeed, busulfan has an early impact on undifferentiated spermatogonia. Five days after busulfan treatment, a decrease of more than 99% of undifferentiated spermatogonia was observed (Supplementary Figure S1), leading to a major loss of other cell types between 2 and 4 weeks post-treatment. Then, the number of undifferentiated spermatogonia was restored to a number similar to the pre-treatment number; the time required for recovery of the number of undifferentiated spermatogonia is dose-dependent [19]. Our results are consistent with this observation. The effect of busulfan is indeed transient; once the number of undifferentiated spermatogonia is restored, spermatogenesis resumes, and the number of cells in the seminiferous tubule and fertility return to normal, although the recovery time is dose-dependent [19]. Regarding the mechanisms at the testicular level, the capacity of germ cells to recolonize is dependent on the population of spermatogonia. Our results highlight the impact on key genes of undifferentiated germ cells. Indeed, using the THY1 + cell sorting of spermatogonia in adult mouse testis, decreased mRNA accumulation of *Thy1*, *Plzf*, *Gfra1*, and *Fgfr2* was observed in the spermatogonia isolated from mice exposed to Bu compared to the control animals (Figure 6B).

Within the testis, there are two subpopulations of undifferentiated spermatogonia, Neurogenin 3 (*NGN3*)-negative cells, which show high stem cell potential, and *NGN3*+ cells, which show high differentiation potential [28]. The transcription factor *NGN3* is expressed in undifferentiated spermatogonia [29] and is required for the entry into the differentiation of *SSC*s [30]. Undifferentiated spermatogonia expressing *Ngn3* therefore do not contribute to *SSC*s; however, after spermatogonial depletion (induced by busulfan treatment), these cells are able to restore the stem cell pool, suggesting that the *NGN3* + cells have been “reprogrammed” into *SSC*s [31]. It appears that *Dmrt1* is required to replenish *SSC*s after germ line depletion [31]. The removal of *Dmrt1* from *NGN3*-positive germ cells was demonstrated to block the replenishment of Id4-GFP-positive *SSC*s and the recovery of spermatogenesis after busulfan treatment. Moreover, it has also been shown that depletion of *Miwi2*-expressing cells results in a transient impact on testicular homeostasis, with this population behaving strictly as transit-amplifying cells under homeostatic conditions. However, upon injury, *Miwi2*-expressing cells are essential for the efficient regenerative capacity of the testis and display facultative stem activity in transplantation assays [32]. In summary, the mouse testis adopted a regenerative strategy to expand stem cell activity by incorporating a transit-amplifying population to the effective stem cell pool, thus ensuring rapid and efficient tissue repair. Interestingly, the present results show that, during a short-term period (1 week), Bu exposure was associated with a decreased expression of *Neurog3* and *Miwi2*, markers of progenitor spermatogonia (Figure 6B). Note that no statistical impact was observed for *Id4*, a main marker of *SSC*s (Figure 6B). This might explain how the germ cell lineage could progress through *SSC*s, resulting in regeneration and recolonization of the seminiferous epithelium.

## 4. Conclusions

The results of this study suggest that Bu treatment may initially impact both *SSC*s and progenitor spermatogonia. However, it is interesting to note that *Id4* expression was less affected, which may partially explain how the germ cell lineage can reemerge in time. Thus, this mechanism could represent a way to minimize the impact of chemodrugs on key *SSC* genes, such as ID4, in order to stimulate their regenerative capacities. However, it still remains to be determined if the differentiated germ cells are of an appropriate quality to produce normal progeny and without the development of pathologies.

In view of our experimental approach, it is important to keep in mind that this single-molecule approach is the first step to better understanding the chemosensitivity of germ cells during chemotherapy treatment. However, it is now well recognized that cancer treatments are more effective when chemotherapy molecules are administered in combination. This strategy of cocktails of drugs is widely used to improve the treatments for patients. Indeed, combinations of molecules are known to improve the selectivity of targets and, thus, to prevent the appearance of resistance to treatment. In anticancer drug cocktails, the different chemotherapeutic molecules may work in the same or different pathways to obtain synergistic, additive, and potentiating effects.

It is clear that chemotherapy cocktails must have differential effects compared to exposure to single molecules. We now need to explore whether the identified effects of Bu and potential downstream signaling targets could be extrapolated to protocols using chemotherapy cocktails. Thus, there is still a significant amount of research needed to identify if key markers of *SSC*s, such as ID4, could be associated with the impact of chemodrugs, so as to target them for preserving stem cell populations, which could allow for germ cell regeneration following anticancer treatment.

## Data Availability

Data are available upon request to the corresponding author; for the RNAseq data, the accession number for the RNAseq data reported in this paper is GSE164734.

## Supplementary Materials

The following are available online at https://www.mdpi.com/article/10.3390/cells10092403/s1. Figure S1: The number of PLZF-positive cells per seminiferous tubules in testes treated with vehicle or busulfan (5 days after treatment). Table S1: List of genes decreased by Bu versus the vehicle. Table S2: List of genes increased by Bu versus the vehicle. Table S3: Gene ontology. Table S4: List of genes with potential E2F4 binding sites. Table S5: List of genes potentially regulated by SIN3A.
