# Supplementary material for: Analysis of the Reversible Impact of the Chemodrug Busulfan on Mouse Testes
Source: Cells. 2021 Sep 13;10(9):2403. doi: 10.3390/cells10092403 (PMC8472455; doi:10.3390/cells10092403)
Supplement: Supplementary file 1 [file cells-10-02403-s001.zip › cells-1315888_Supplementary Material/cells-1315888_supplementary material.pdf]

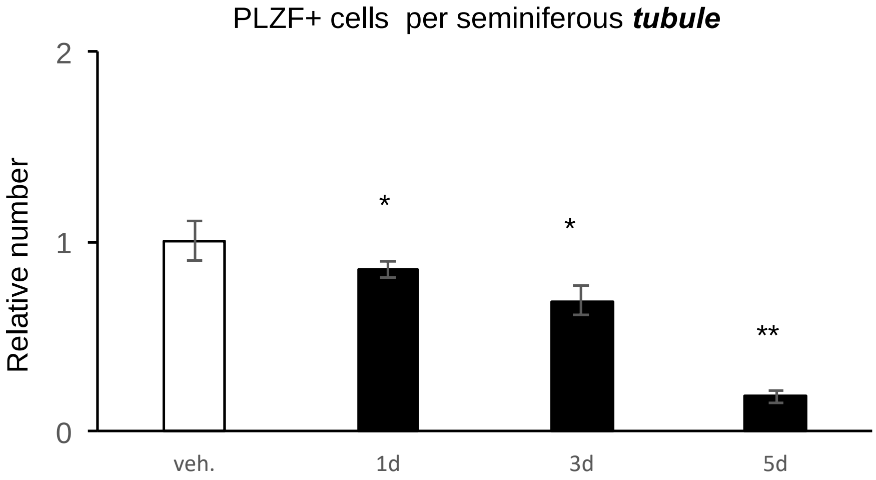


**Figure S1.** Quantification of the relative number of PLZF positive cells per seminiferous tubules in *Wt* testis treated with busulfan (1 to 5 days after treatment). The mean number of PLZF+cells observed in *Wt* males treated with Bu was arbitrarily set at 100 for comparison with the number of G9A+ cells observed one and two weeks after treatment.
